# Guaranteeing Isochronous Control of Networked Motion Control Systems Using Phase Offset Adjustment

**DOI:** 10.3390/s150613945

**Published:** 2015-06-12

**Authors:** Ikhwan Kim, Taehyoun Kim

**Affiliations:** Department of Mechanical and Information Engineering, University of Seoul, 163 Seoulsiripdae-ro, Dongdaemun-gu, Seoul 130-743, Korea; E-Mail: ihkim@uos.ac.kr

**Keywords:** networked motion control systems, isochronous control, phase offset adjustment, EtherCAT, open source software

## Abstract

Guaranteeing isochronous transfer of control commands is an essential function for networked motion control systems. The adoption of real-time Ethernet (RTE) technologies may be profitable in guaranteeing deterministic transfer of control messages. However, unpredictable behavior of software in the motion controller often results in unexpectedly large deviation in control message transmission intervals, and thus leads to imprecise motion. This paper presents a simple and efficient heuristic to guarantee the end-to-end isochronous control with very small jitter. The key idea of our approach is to adjust the phase offset of control message transmission time in the motion controller by investigating the behavior of motion control task. In realizing the idea, we performed a pre-runtime analysis to determine a safe and reliable phase offset and applied the phase offset to the runtime code of motion controller by customizing an open-source based integrated development environment (IDE). We also constructed an EtherCAT-based motion control system testbed and performed extensive experiments on the testbed to verify the effectiveness of our approach. The experimental results show that our heuristic is highly effective even for low-end embedded controller implemented in open-source software components under various configurations of control period and the number of motor drives.

## 1. Introduction

Cyber Physical Systems (CPS) are physical and engineered systems whose operations are monitored, coordinated, controlled and integrated by a computing and communication core [1]. Motion control systems (MCS), one of typical CPS applications, have been widely used in various industrial fields such as packaging, semiconductor manufacturing, and production machinery [2–4], and are becoming important driving components for the next industrial revolution. A typical MCS consists of computational units such as a motion controller and motor drives, and physical components such as actuators and sensors, that are tightly coupled and collaborate with each other in synchronized manner. In a recent decade, MCS are facing a steady but fundamental change, *i.e.*, the introduction of real-time Ethernet (RTE) as the communication core, replacing conventional fieldbuses [5].

Networked MCS have stringent real-time constraints. Two of the most important are the bounded end-to-end actuation delay and its deviation. The end-to-end actuation delay refers to the time interval from the dispatch of control commands at the controller to the corresponding actuation at a motor drive. Actually, the maximum value of this delay limits the achievable minimum cycle time (MCT) at the controller. In general, the shorter the end-to-end actuation delay is, the higher the *precision* of single-axis motion becomes. As the term *cycle* implies, the transfer of actuation control message should also be isochronous while keeping the jitters of successive control message delivery intervals as small as possible. The deviation in the actuation delay is the time difference between the earliest and the latest actuation at different motor drives in the same control cycle. Similarly, the smaller the actuation deviation is, the higher the *synchronicity* of multi-axis coordinated motion becomes.

With an RTE communication network, previous works [6–8] show that careful message scheduling and hardware-based frame switching can come up with the real-time constraints mentioned above at the communication level. However, as pointed out in [9,10], such high precision and synchronicity should be validated by a thorough analysis of the networked process delay, which includes the time for in-controller processing, message delivery, and local handling by each motor drive. Although the clock-driven synchronization method such as distributed clock (DC) in EtherCAT [11] can hide the adverse effect on isochronous control caused by the deviation of the end-to-end actuation delay, its performance also relies on the time-deterministic end-to-end message delivery. Employing hard real-time commercial OS or dedicated hardware optimization may also be alternative solution for this problem. However, it would be a high-cost, time-consuming and error-prone job for engineers who have only limited domain-knowledge. Hence, in this paper, we propose a simple and efficient heuristic to guarantee isochronous control message delivery for higher precision and synchronicity of networked motion control in a systematic manner.

In this paper, we first formulate the isochronous control property required by target motion control systems. The property may not hold due to non-deterministic behavior of software in the motion controller in spite of the determinism provided by the underlying real-time communication technologies. To come up with the problem, we propose a simple and efficient phase offset adjustment heuristic to provide deterministic end-to-end isochronous control. In realizing the proposed heuristic, we customized an open-source, integrated development environment (IDE) to determine the proper phase offset in pre-runtime and applied the phase offset value to the runtime code of the motion controller in a systematic manner. By using a pre-runtime analysis, we eliminate possible interferences with real-time, deterministic message transfer at runtime. On an EtherCAT-based motion control system test-bed, we evaluated the jitters of the intervals between successive control frames observed at the motor drives for varying number of motor drives and control cycle. The experimental results show that the proposed heuristic can greatly reduce the deviation of intervals between successive control frame arrivals for various system configurations, even for the low-performance embedded controller. It is noteworthy that simultaneous actuation among different motor drives using clock-driven synchronization also relies on deterministic control message arrival time at the drive. Hence, our heuristic can also be applied profitably in determining the safe delay value of global clock event at each motor drive.

The rest of this paper is organized as follows. In Section 2, we review the background to networked motion control systems and present related works. Section 3 formulates the problem addressed in this paper and describes our heuristic to come up with the problem. We evaluate the effectiveness of the proposed approach in Section 4. Finally, Section 5 concludes this paper.

## 2. Background and Related Works

### 2.1. Real-Time Requirements for Modern Motion Control System

**Figure 1:**
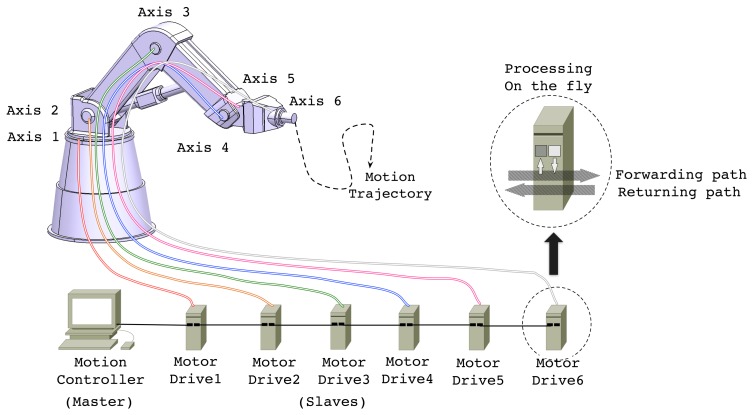
An example of EtherCAT-based motion system with 6-DOF robot application.

In a modern MCS considered in this paper, a motion controller and a number of motor drives, which are interconnected through industrial communication links, cooperate with each other in a synchronized manner. Figure 1 illustrates an example of such a configuration, a 6 degrees-of-freedom (DOF) industrial robot, of which components are interconnected through an EtherCAT network in line topology. In the robot system, the motion controller periodically generates control messages containing the commands of target position or velocity and transmits them to the motor drives. On receiving the control information, each motor drive operates its control loop and actuates the corresponding axis. The motor drives are also responsible for reporting status, *i.e.*, current position and velocity, acquired from the associated sensors. By using the feedback information from the motor drives, the motion controller computes control command. In doing so, the operation of motor drives including actuation and sensing should also be carefully coordinated or synchronized with others to make the tool point accurately follow the desired motion trajectory. From the industrial robot example shown in Figure 1, we can derive real-time constraints generally imposed by the modern MCS.

First, the motion system must complete its operation regarding control command computation and data exchange between the controller and all the drives within a pre-defined cycle time for every control cycle. Although the desired cycle time depends on the application requirements, it is known that the cycle time less than 1 ms is needed for the general motion control applications [12]. For the accurate motion conforming to the designated motion profile, it is also crucial to guarantee that successive actuation and sensing operations performed by a motor drive should be performed at fixed intervals, *i.e.*, *isochronous*. Furthermore, the achievable MCT in the system should be as small as possible to achieve higher-level of precision in the motion in many cases. As mentioned in Section 1, although the introduction of RTE communication network has been greatly contributed to achieve short and deterministic message relay, a holistic analysis and optimization of other processing overhead including in-controller processing delay and drive-local delays should also be considered to guarantee bounded end-to-end control delay and isochronous operations.

Second, for the higher accuracy of coordinated motion, each drive must perform actuation and sensing being accurately synchronized with other drives. A common approach to cope with the requirement is to use a global clock across the networked control system. Assuming that the local clocks at different drives are properly synchronized to a global reference clock, an actuation and sensing task in drives can be invoked by the autonomous interrupt that is generated synchronous to the global clock. Thus, the actuation deviation among different drives can be bounded to negligibly small value. Generally, the clock events are generated with a predefined shift time from the beginning of the control cycle. However, it is often regarded trivial or even ignored that, to properly handle the fresh commands from the controller, the clock events must occur after the control packets from the controller have been received. For this reason, it is very important to estimate the end-to-end delay observed at the drives to determine a safe clock event shift time. From a practical viewpoint, the deviation of the end-to-end delay of control command transmission primarily depends on the fluctuation in controller processing delay because other delay factors in link-level communication latency and drive-local operations are rather deterministic as presented in previous works [8–10]. Hence, this paper focuses on guaranteeing isochronous control by keeping the jitters of successive control message transfer as small as possible at the controller level.

### 2.2. EtherCAT Synchronization Methods

Among the RTE networks, we adopt EtherCAT [13] as the communication core of our target system due to its desirable features such as deterministic communication delay, high-speed message relaying, efficient synchronization feature based on global clock, flexible topology, and cost-effectiveness [5]. In a typical motion control system configuration as shown in Figure 1, the master, *i.e.*, the motion controller, periodically generates and transmits motion control commands in the form of EtherCAT frame to the slaves, *i.e.*, motor drives. Along the forwarding path, every slave in an EtherCAT network relays control frames between the input and output ports *on-the-fly* using a dedicated switching hardware. Once an EtherCAT frame arrives at the end of the network, it returns to the controller along the returning path updating the contents acquired from the associated sensors on-the-fly.

**Figure 2:**
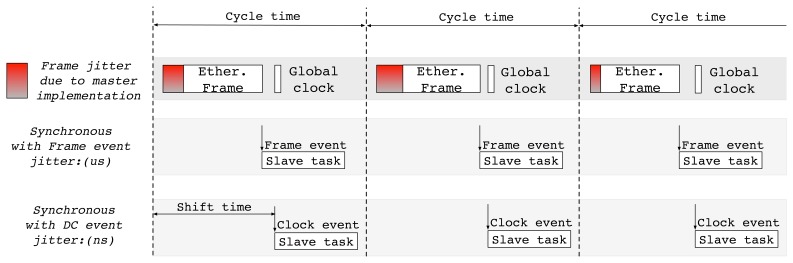
Two synchronization modes in the EtherCAT slave.

EtherCAT also provides two kinds of synchronization events, *i.e.*, frame events and clock events, for the coordinated operations of multiple slaves [9]. As shown in Figure 2, a local slave task, which is responsible for actuation or sensing, can be synchronized with these events. The frame-driven control process may often experience relatively large deviation due to the variation in in-controller processing time. When a slave task requires a higher-degree of cycle precision with low deviation, a globally synchronized clock should be used. The slave task may take actions based on the autonomous interrupt that is generated with the global clock, referred as the Distributed Clock (DC), and therefore, the deviation of control process can be reduced by up to a few nanoseconds [11].

### 2.3. Related Works

Jitter compensation to avoid performance degradation and instability of control systems has already been studied in traditional real-time control literature. Marti *et al.* categorized three types of jitters in a control loop, which consists of sampling, control computation and actuation, and proposed a control parameter adjustment to compensate for those jitters [14]. Buttazo and Cervin presented three different approaches for reducing the jitter in control systems and evaluated their performances by simulation [15]. These researches have limitations in that they only considered a single, self-contained control system, which is far from modern control system architecture with distributed control nodes connected through communication links, and the efficiency of proposed methodologies has just been proved by simulation.

One of the major performance indicator for networked control systems has been the achievable MCT. Early works in [6–8] formulated the network-level delay components of EtherCAT network to find an MCT according to the control frame size. However, from practical viewpoints, a holistic end-to-end delay and jitter model considering other factors such as in-controller delay and drive-internal operations is required to find a viable MCT. Recent studies to guarantee deterministic operation of motion controller have been presented in two folds. Studies in [16,17] proposed highly customized solutions for the applications which require very short cycle time, *i.e.*, under 1 ms. With the customized hardware and the use of commercial real-time OS, they optimized the operation of EtherCAT protocol stack and presented the achievable MCT under 100 μs including controller internal delay. On the other hand, with the increasing use of open source software (OSS), there are approaches to adopt OSS solutions in realizing real-time motion control systems [9,18–20]. In common, they constructed a real-time motion controller with real-time Linux and open source EtherCAT protocol stack on top of commercial-off-the-shelf (COTS) hardware. The performance evaluation results of them show that OSS-based control systems can also provide very short control cycle with small jitter.

Recent studies have also begun to address a holistic end-to-end delay model of entire motion control system considering in-controller delay or drive-local operations [9,10,21]. Kim *et al.* considered the internal operation of motor drives, but they have considered the message release interval of controller as constant value [10]. Sung *et al.* measured the in-controller processing delay in order to carry out the end-to-end delay analysis where EtherCAT synchronization methods are used [9]. Lee *et al.* proposed a simulation framework to find an optimal phase of distributed nodes with respect to the end-to-end delay and the actuation jitters [21]. However, these works lack any consideration of actual workload [9,21] or do not consider the message release jitter caused by the motion controller [9,10,21].

## 3. Phase Offset Adjustment for Isochronous Control: A Tool-based Heuristic Approach

### 3.1. System Model and Problem Statement

In this paper, we consider an MCS which consists of a motion controller and *N* (≥1) homogenous motor drives interconnected through EtherCAT communication link in line topology. Generally, a control loop on the controller is assumed to be a periodic activity, of which parts consisting of sampling, computation, and actuation. The control loop can be implemented as a single task or a set of subtasks where each subtask performs one or more parts of a control loop [14]. For simplicity, this paper assumes a single task implementation where the task performs the parts sequentially. We also assume that the control task is an application task with the highest priority among the application-level tasks. Therefore, the execution of the control task can be delayed or preempted only by kernel-level system maintenance services and I/O interrupt handling. Since we assumed homogenous motor drives, the delay caused by the drive-local operations for each drive can also be assumed to be identical.

**Figure 3:**
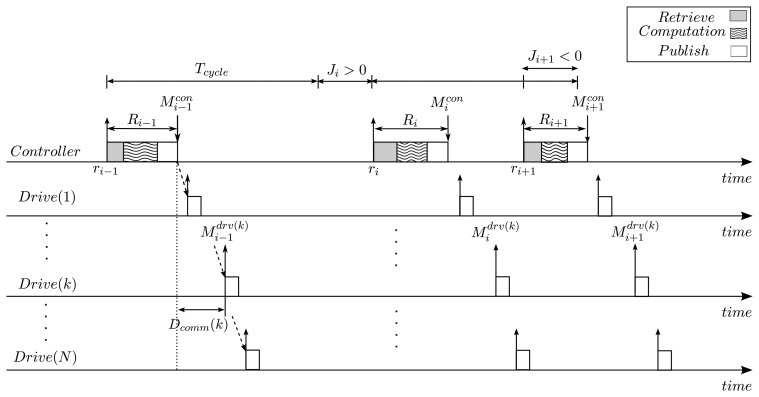
System model.

As mentioned above, the control task, called *τ*, performs periodically a control sequence, which consists of *retrieve*, *computation* and *publish* operations. Note that the terms *retrieve* and *publish* are used instead of sampling and actuation, respectively, to emphasize that the operations are performed for motor drives distributed over the industrial communication link. In the *retrieve* phase, the control task reads the status information including current position and velocity reported by the motor drives during the previous control cycle. Using the information acquired in the *retrieve* phase, the control task checks if the motors are being moved properly according to the planned motion trajectory and, if not, generates correction commands in the *computation* phase. Finally, in the *publish* phase, the controller transmits motion commands to the motor drives. Figure 3 depicts the timing parameters deal with in our model.

We now consider the timing properties of the control task *τ*. The control task is released periodically at fixed intervals, called *T_cycle_*, and the *i*-th instance of *τ* is denoted by *τ_i_*. In traditional real-time literature, the task instances are assumed to be released strictly on time, and thus the release time of *τ_i_* is defined as *r_i_* = (*i* − 1) · *T_cycle_*. However, task instances may often experience arbitrary release jitter, denoted by *J_i_*, due to timer resolution and interfering kernel-level operations. Then, the actual release time of *τ_i_* is given by ∀*i* ≥ 1*, r_i_* = *r_i−_*_1_ + *T_cycle_* + *J_i_*, where *r*_0_ = 0 and *J*_0_ = 0. As shown in Figure 3, the release jitter *J_i_* can be other than 0, which means that the task instance was released earlier, *J_i_* < 0, or later , *J_i_* > 0, than the designated release time. The response time of *τ_i_, R_i_* is the difference of its finishing time and its release time. The control task *τ* completes its execution when *the publish* phase is finished. Then, if we denote the finishing time of *τ_i_* as $M_{i}^{\text{con}}$, $M_{i}^{\text{con}}$ is given by:

(1) $$\begin{array}{r} M_{0}^{\text{con}}=r_{0}+R_{0}=R_{0},\left(r_{0}=0,J_{0}=0\right)\\ M_{1}^{\text{con}}=r_{1}+R_{1}=\left(r_{0}+T_{\text{cycle}}+J_{1}\right)+R_{1}=\left(T_{\text{cycle}}+J_{1}\right)+R_{1}\\ M_{2}^{\text{con}}=r_{2}+R_{2}=\left(r_{1}+T_{\text{cycle}}+J_{2}\right)+R_{2}=\left(2\cdot T_{\text{cycle}}+J_{1}+J_{2}\right)+R_{2}\\ \cdots \cdots \\ M_{i}^{\text{con}}=r_{i}+R_{i}=i\cdot T_{\text{cycle}}+\sum_{n=1}^{i}J_{n}+R_{i} \end{array}$$

For the holistic approach to the problem in this paper, we now consider the timing properties observed at the motor drives. Let $M_{i}^{\text{drv}\left(k\right)}$ is the time when the *i*-th control message arrives at the *k*-th motor drive where *k* (1 ≤ *k* ≤ *N*) means that the drive is *k*-th nearest from the controller. Then, we can express $M_{i}^{\text{drv}\left(k\right)}$ as:

(2) $$M_{i}^{\text{drv}\left(k\right)}=M_{i}^{\text{con}}+D_{\text{comm}}\left(k\right)$$

where $M_{i}^{\text{con}}$ and *D_comm_*(*k*) refer to the time when *i*-th motion message is released at the controller and the message communication delay to *k*-th drive, respectively. *D_comm_*(*k*) is computed by *D_comm_*(*k*) = *D_link_* + *k* · (*D_relay_* + *D_prop_*). As suggested in [8], the link transmission delay *D_link_* can be easily computed when the size of message payload and the link capacity are known *a priori*. Again, *D_relay_* and *D_prop_* refer to the message frame relaying delay at each motor drive and the cable propagation delay between consecutive drives, respectively According to previous works [8,9], the sum of *D_relay_* and *D_prop_* can be treated as a small constant, e.g., 1 μs, thanks to the on-the-fly frame relay feature of EtherCAT. Therefore, *D_comm_*(*k*) is also treated as constant value for a fixed *k* in this paper.

To guarantee isochronous actuation, it is required that the time differences between two consecutive motion message arrivals observed at the *k*-th motor drive should be equal to *T_cycle_* as expressed in Equation (3).

(3) $$M_{i+1}^{\text{drv}\left(k\right)}=M_{i}^{\text{drv}\left(k\right)}+T_{\text{cycle}}$$

Since we treat *D_comm_*(*k*) as constant, Equation (3) is reduced to:

(4) $$\left(M_{i+1}^{\text{con}}-M_{i}^{\text{con}}\right)=T_{\text{cycle}}$$

From Equations (1) and (4), it follows that the condition to guarantee isochronous control is alternatively given by:

(5) $$\forall_{i}\geq 1,J_{i}+\left(R_{i}-R_{i-1}\right)=0$$

### 3.2. Proposed Heuristic

As mentioned above, the task release jitter *J_i_* and the differences of response time of consecutive task instances (*R_i_* − *R_i−_*_1_) should be zero to guarantee isochronous control. However, it is very difficult to keep these values zero in practice even when we use commercial hard real-time OS and highly customized hardware. Instead, we aim to keep the condition expressed in Equation (4) be approximately met, that is, to keep the maximum variation between two consecutive message release time, $\left(M_{i+1}^{\text{con}}-M_{i}^{\text{con}}\right)$, as small as possible.

Strictly speaking, $M_{i}^{\text{con}}$ should be the time when network interface controller (NIC) of the motion controller starts to transmit the control command packet, which can hardly be controlled. Instead, we can define $M_{i}^{\text{con}}$ as the time when the *i*-th command packet is copied to the FIFO buffer in the NIC. However, it requires cumbersome porting job when the underlying OS and/or NIC hardware is changed. Hence, for higher portability of our heuristic implementation, we propose to control the starts times of the *publish* phase in the control task instances. Note that, hereinafter, $M_{i}^{\text{con}}$ refers to the start time of the *publish* phase in *τ_i_* unless otherwise specified. It follows that the computation of $M_{i}^{\text{con}}$ is rewritten as:

(6) $$M_{i}^{\text{con}}=i\cdot T_{\text{cycle}}+\sum_{n=1}^{i}J_{n}+R_{i}^{\text{comp}}$$

where $R_{i}^{\text{comp}}$ represents, for the *i*-th task instance, the time difference of its *computation* phase finishing time and its release time.

In a straightforward implementation of the control task, *the publish* phase starts to execute right after the completion of the *computation* phase. However, in our heuristic, the *publish* phase of task instances is forced to be executed with a proper offset from their designated release time. Suppose that a proper offset Φ*_p_* is known *a priori*, the first task instance *τ*_0_ starts its *publish* phase with the phase offset Φ*_p_* from its release time, and *the publish* phases of successive task instances start with a fixed interval *T_cycle_*. Then, the adjusted start time of *publish* phase $M_{i}^{{\text{con}}^{\prime }}$ is give by:

(7) $$\forall_{i}\geq 1,M_{i}^{{\text{con}}^{\prime }}=i\cdot T_{\text{cycle}}+M_{0}^{{\text{con}}^{\prime }}$$

where $M_{0}^{{\text{con}}^{\prime }}=r_{0}+\Phi_{p}$.

We now explain the way how to determine the proper phase offset Φ*_p_*. In determining the phase offset, we should consider the following constraints:

- (1) For all the control cycles, the condition $\left(M_{i}^{{\text{con}}^{\prime }}>M_{i}^{\text{con}}\right)$ should be met in order not for the controller to try to transmit out-of-date control command because the *computation* phase may not be completed at the time the controller begins to transmit the control command. The phase offset value Φ*_p_* satisfying this constraint becomes the lower bound of Φ*_p_*, denoted by $\Phi_{p}^{-}$.
- (2) Any two consecutive control cycles should not overlap with each other. If this constraint is violated, the next control cycle should wait for the arrival of fresh feedback information from the drives. Such a situation may occur when the adjusted phase offset becomes too large. Then, the phase offset value Φ*_p_* satisfying this constraint is the upper bound of Φ*_p_*, denoted by $\Phi_{p}^{+}$.
- (3) For safety, the condition $\left(\Phi_{p}^{-}<\Phi_{p}^{+}\right)$ must be satisfied. Otherwise, it means that there may not be enough time for all the required operations to complete in a control cycle.

**Figure 4:**
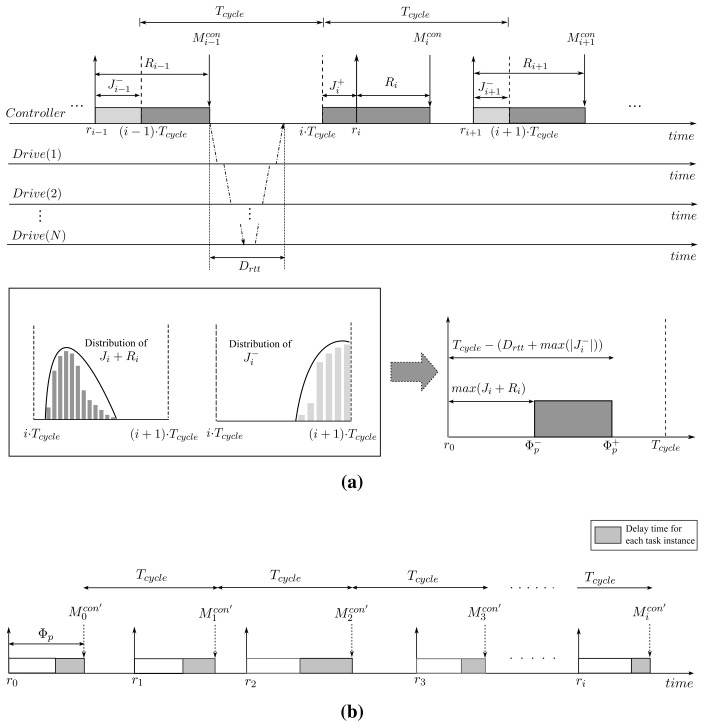
Our proposed heuristic. (**a**) Determine phase offset Φ*_p_* for the *publish* phase; (**b**) Execution of motion control task with phase offset Φ*_p_*.

Since it is very difficult to determine $\Phi_{p}^{-}$ satisfying Constraint (1) theoretically for randomly distributed *J_i_* and $R_{i}^{\text{comp}}$, we estimate $\Phi_{p}^{-}$ with the *J_i_* and $R_{i}^{\text{comp}}$ values obtained empirically through a pre-runtime analysis. In determining $\Phi_{p}^{+}$ satisfying Constraint (2), a certain amount of time should be reserved between the control message release time and the release time of next control period. The reserved time amounts to the time taken to deliver the control commands to all the drives and convey the feedback information from the drives under the worst-case release jitter scenario. The worst-case release jitter scenario means that the next task instance is released earlier with maximum task release jitter *max* $\left(\left|J_{i}^{-}\right|\right)$. Let *D_rtt_* denote the round-trip time taken for the communication, then we can compute *D_rtt_* by *D_rtt_* = (*2N −* 1) · *D_relay_* + *2N* · *D_prop_* + *D_link_*. In summary, the safe bound of phase offset Φ*_p_* satisfying the constraints above is expressed as:

(8) $$\Phi_{p}=\left[\Phi_{p}^{-},\Phi_{p}^{+}\right]=[\max\left(J_{i}+R_{i}^{\text{comp}}\right),T_{\text{cycle}}-\left(D_{\text{rtt}}+\max\left(\left|J_{i}^{-}\right|\right)\right)],i\geq 1$$

where *max* $\left(\left|J_{i}^{-}\right|\right)$ represents the maximum release jitter for the task instance among the task instances released earlier than the designated release time. Figure 4 outlines the discussion in this section.

### 3.3. Implementation of Our Heuristic

As described earlier, our goal is to minimize actuation jitters observed at the motor drives by keeping the time intervals of successive control message transfer by the controller at fixed intervals. For this purpose, we devised a heuristic to delay control message transfer time, *i.e.*, message publish time, based on the estimation of the worst-case start time of the *publish* phase. In realizing our heuristic, we aim to avoid modifying kernel-level code or using customized hardware in order to save engineering efforts of engineers who have domain-knowledge only. Hence, we used a pre-runtime analysis integrated with an open-source IDE for motion applications, called Beremiz [20], to estimate the safe phase offset of the *publish* phase, Φ*_p_*. The runtime executable image generated by Beremiz is composed of motion application codes, OS-dependent task management stubs, and EtherCAT communication codes.

**Figure 5:**
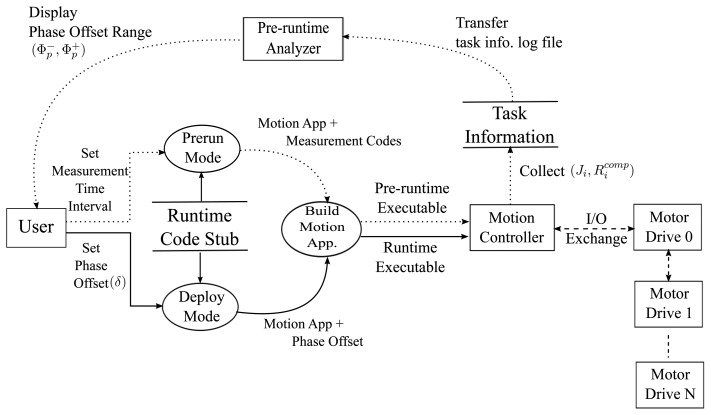
Execution flow of pre-runtime analysis.

**Figure 6:**
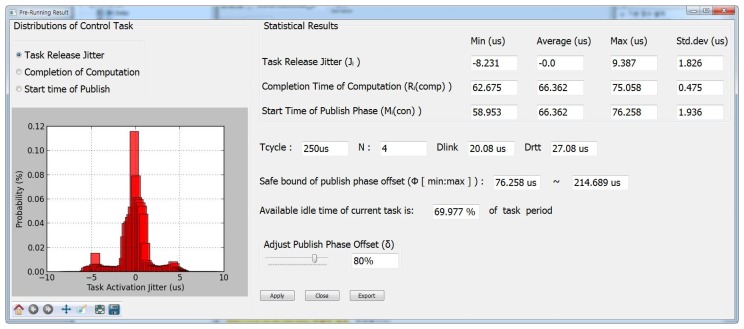
User interface of pre-runtime analyzer.

In order to obtain the information for determining the phase offset Φ*_p_* during the pre-run cycles, we first modified the task main body generation part for the pre-run mode to insert time measurement code using timer API supported by target OS, e.g., *rt_timer_read* API in Xenomai. Figure 5 depicts the execution flow of our pre-runtime analysis. In the pre-run cycles, the motion runtime code executes for the time intervals, which is configurable by users, and the information such as release jitter *J_i_* and response time of *computation* phase $R_{i}^{\text{comp}}$ is collected. Then, the collected information is analyzed and displayed by the pre-runtime analyzer. As shown in Figure 6, the analyzer suggests the range of safe phase offset values computed by Equation (8). Note that the phase offset value represented as the percentile ratio normalized with respect to the control cycle, denoted by *δ_p_*, *i.e.*, Φ*_p_* = *δ_p_* · *T_cycle_*. In addition, finally, once the user choose the phase offset value, the offset is applied to the final runtime code for the control task. Listing 1 describes the pseudo code for the control task body generated by the Beremiz IDE.

#### Listing 1. Pseudo code of control task with proposed heuristic.

**while**
(! PLC_shutdown) 
retrieving sensed values; 
computing motion command; **if**
first_iter:  /* Delay transmission time at first task instance */  
time2transmit = first_release_time + $\delta$ * TSK_PERIOD; **else**  /* Next transmission time is determined by   sum of previous transmission time and task period */  
time2transmit += TSK_PERIOD; **endif** 
now = get current system time; /* Waiting for the time to transmit */ **while**
(now < time2transmit)  
now = get current system time; **endwhile** 
time2transmit = now;  
publishing motion command; 
wait until next task activation;**endwhile**

## 4. Performance Evaluation

In this section, we present experimental results to verify the effectiveness of our proposed heuristic. We performed a series of experiments in terms of the frame inter-arrival times observed at motor drives, $T_{i}^{\text{drv}\left(k\right)}=M_{i}^{\text{drv}\left(k\right)}-M_{i-1}^{\text{drv}\left(k\right)}$, for various configurations, *i.e.*, different types of controller, the control period *T_cycle_*, and the number of motor drives *N*. A safe and reliable phase offset factor *δ_p_* was obtained by pre-runtime analysis on a testbed.

### 4.1. Experimental Setup

**Figure 7:**
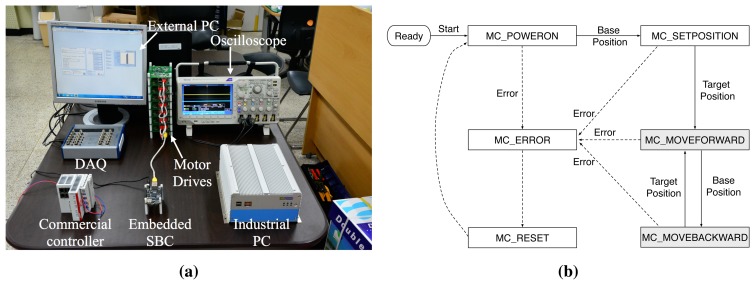
Experimental setup. (**a**) Motion control system testbed; (**b**) State transition of PTP motion application.

The performance evaluation was conducted on an EtherCAT-based MCS testbed which is compromised of a motion controller, multiple homogeneous motor drives, and external measurement equipments as shown in Figure 7a. For the comparison of different implementations of motion controller, we used three different types of motion controller: a high-end industrial PC (IPC), a low-end embedded single board computer (SBC) board, and a commercial EtherCAT master controller. The IPC and embedded SBC were constructed solely from the open-source components such as Xenomai real-time patch for Linux [22] and IgH EtherCAT master stack [23]. Table 1 summarizes the details of our testbed. As the test motion application, we used a point-to-point (PTP) movement application in which each motor drive repeats to move from base position to target position and vice versa. Figure 7b depicts the state transition of the test application.

In order to reduce potential interferences caused by kernel-level tasks, open-source based controllers, e.g., IPC and embedded SBC, were configured to start with single-user mode and operate without X-windows desktop manager. In addition, the modified network device driver and NEON floating point unit (FPU) compiler option proposed in our previous work [24] are used for embedded SBC as the platform optimization. For the motor drive platform, we chose TI industrial communication engine (ICE) board and configured the drives to use identical process data object (PDO) data set for cyclic synchronous position (CSP) mode operation. The frame arrival time observed at the motor drives, $M_{i}^{\text{drv}\left(k\right)}$, was measured by logging GPIO output signals generated at the time when an EtherCAT frame arrives. The GPIO signals were collected by using an external data acquisition (DAQ) board with *ns* resolution timestamp. The statistical analysis on the measured samples was conducted using a LabVIEW application.

### 4.2. Analysis of the Behavior of Motion Control Task

As described in Section 3.2, we need to obtain pre-runtime analysis results to determine a safe phase offset Φ*_p_* for the *publish* phase. Recall that the lower bound of Φ*_p_*, $\Phi_{p}^{-}$, is determined by the worst-case $\left(J_{i}+R_{i}^{\text{comp}}\right)$ and the upper bound of Φ*_p_*, $\Phi_{p}^{+}$, is determined by the terms *max* $\left(\left|J_{i}^{-}\right|\right)$ and *D_rtt_*, respectively. Thus, we measured the *J_i_* and $R_{i}^{\text{comp}}$ values of the control task *τ* and analyzed them on open-source based controllers.

Table 2 shows the measurement results of *J_i_*, $R_{i}^{\text{comp}}$, and $M_{i}^{\text{con}}$ as we vary the length of control cycle *T_cycle_* and the number of motor drives *N*. The $M_{i}^{\text{con}}$ values, which are the start times of *publish* phase without heuristic, were also measured and analyzed for the comparison with the results of our heuristic later in Section 4.3. For each experiment, we conducted the measurement for 5 min, of which collected data amounts to 1,200,000 samples with *T_cycle_* = 250 μs for instance. In the results, Δ denotes the absolute difference between the maximum and minimum value for each term.

According to Table 2, when we use high-end IPC controller, the task release jitter *J_i_* values are quite stable for varying *T_cycle_* and *N*. By contrast, *J_i_* for low-end embedded SBC tends to increase as we increase the control cycle *T_cycle_*. It is considered that the cache pollution due to other background tasks and kernel activities is enlarging the release jitter of control task as pointed out in previous works [9,19]. As for $R_{i}^{\text{comp}}$, its average and deviation depend on both *T_cycle_* and *N* when we use embedded SBC. Regardless of the controller used, the average of $R_{i}^{\text{comp}}$ increases with respect to *N*. This is because the feedback data size is linearly increased with *N* and the motion computation is performed for each axis, *i.e.*, drive, and thus the execution time for the *retrieve* phase and *computation* phase is monotonically increased. On the other hand, the deviation of $R_{i}^{\text{comp}}$ using embedded SBC decreases significantly for shorter *T_cycle_* with a fixed *N*. It is considered that, with shorter *T_cycle_*, there are less probabilities for the control task to be exposed to the interferences from kernel-level activities and cache miss handling overhead will also be reduced. As for $M_{i}^{\text{con}}$, IPC and embedded SBC show different characteristics. With IPC controller, the deviation and absolute difference values of $M_{i}^{\text{con}}$ are quite stable for all the experimental configurations. However, with embedded SBC, we observe that the values increase with respect to *T_cycle_* and *N*, which indicates the deviation of $M_{i}^{\text{con}}$ in embedded SBC is affected by both *J_i_* and $R_{i}^{\text{comp}}$.

**Figure 8:**
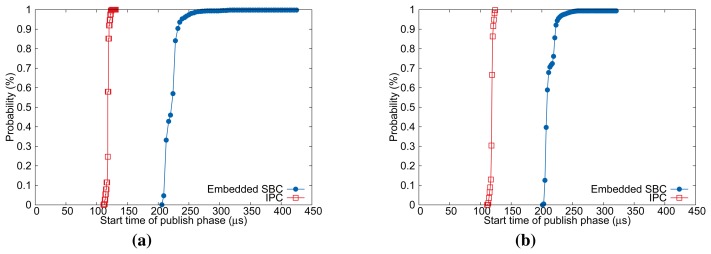
The CDF distribution of start time of *publish* phase in the *T_cycle_*. (**a**) *N* = 8, *T_cycle_*: 1000μs; (**b**) *N* = 8, *T_cycle_*: 500μs.

Figure 8 illustrates the cumulative distribution function (CDF) of measured $M_{i}^{\text{con}}$ values. As mentioned earlier, the sum of *J_i_* and $R_{i}^{\text{comp}}$ determine $M_{i}^{\text{con}}$ and the absolute difference Δ of *J_i_* and $R_{i}^{\text{comp}}$ primarily contributes to the deviation of $M_{i}^{\text{con}}$. This characteristic is more apparent for embedded SBC as shown in Figure 8. In summary, we expect from the pre-runtime analysis results that our heuristic will be more beneficial to low-end embedded SBC with highly fluctuating message release interval.

From the measurement results given in Table 2, we can obtain the range of safe phase offset factor *δ_p_* as given in Equation (8). Since *D_rtt_* is the function of *N* and other parameters are known *a priori*, it can also be easily computed. Table 3 summarizes the phase offset factors normalized with respect to *T_cycle_* when we vary the number of motor drives from 1 to 8 and the control cycle *T_cycle_* from 250 μs to 1000 μs, respectively. It is noticeable that we cannot determine the safe phase offset factor for embedded SBC with *T_cycle_* = 250 μs. This is because the estimated $\Phi_{p}^{-}$ and $\Phi_{p}^{+}$ for the case were 94% and 84%, respectively, which violates Constraint (3) in Section 3.2. It is noteworthy that our pre-runtime analysis can also be used to estimate the achievable MCT for a given configuration.

### 4.3. Frame Inter-Arrival Time Observed at Motor Drives

The objective of our heuristic is to keep the differences between frame inter-arrival times observed at the motor drives, $T_{i}^{\text{drv}\left(k\right)}$, and the control cycle *T_cycle_* as small as possible for deterministic isochronous control. To verify the effectiveness of our approach, we conducted a set of experiments to measure $T_{i}^{\text{drv}\left(k\right)}$ for different controller implementations and compared the results. In each experiment, the measurement was performed for 30 min, for instance, of which collected data size amounts to 3,600,000 samples with *T_cycle_* = 500 μs. Since we used homogeneous motor drives and the switching and propagation delay of frames is very small and bounded, we can regard the values observed at the first motor drive, $T_{i}^{\text{drv}\left(k\right)}$, as the representative case without loss of generality. For performance evaluation, we define additional performance indicators, *ϵ*_1%_ and *ϵ*_10%_. The parameters *ϵ*_1%_ and *ϵ*_10%_ account for the number of intervals between successive frame arrivals, of which length exceeding 1% and 10% with respect to a given *T_cycle_*, respectively.

**Figure 9:**
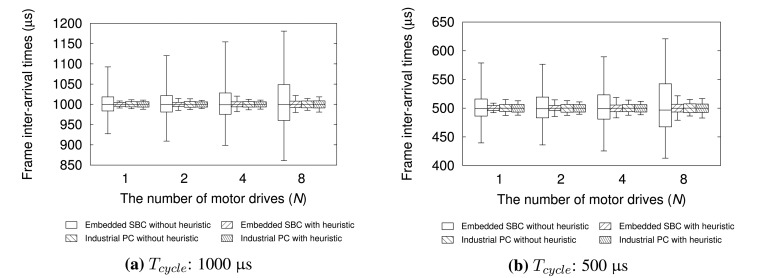
Distribution of frame inter-arrival times for different controller configurations.

We first measured and compared the frame inter-arrival times for various testbed configurations. The configurations were varied according to *T_cycle_, N*, controller hardware types, and whether to use our heuristic or not. For the experiments with our heuristic, we chose the $\delta_{p}^{\max}$ values shown in Table 3. The performance evaluation results are summarized in Table 4 and Figure 9. The boxes in the Figure 9 represent the range in which 99% of measured samples are located. The lower and upper whiskers in the plots refer to the low 0.5% and low 99.5% value shown in Table 4.

As shown in Table 4, IPC greatly outperforms embedded SBC in terms of the deviation of frame inter-arrival time jitters without our heuristic. For all *T_cycle_* and *N* configurations used, IPC controller has zero *ϵ*_10%_ and much smaller *ϵ*_1%_ than embedded SBC. However, after applying our heuristic, we can observe that embedded SBC controller shows performances comparable to IPC for all *T_cycle_* and *N*. For instance, *ϵ*_10%_ is decreased to zero and *ϵ*_1%_ is reduced by up to 89% compared with the case without our heuristic where *T_cycle_* = 500 μs and *N* = 8.

**Figure 10:**
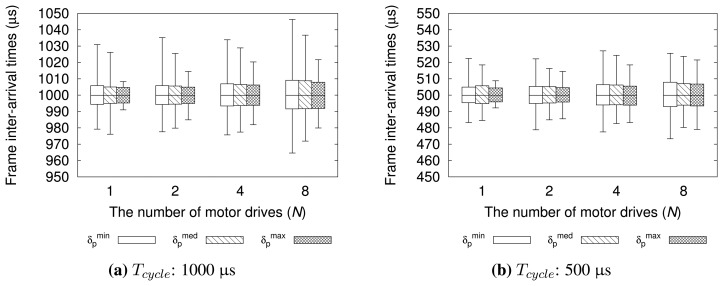
Frame inter-arrival times for different phase offset (*δ_p_*).

We conducted another set of experiments to observe the performances according to *δ_p_* shown in Table 3. Figure 10 illustrates the distributions of frame inter-arrival time for $\delta_{p}^{\min}$, $\delta_{p}^{\mathrm{med}}$, and $\delta_{p}^{\max}$. Compared with the results in Figure 9, our heuristic can greatly reduce the deviation of frame inter-arrival time irrespective of *δ_p_* used where the 99% of frame inter-arrival times have jitters less than ±8 μs for all *N*. However, we can also notice that the advantage of our heuristic is much more outstanding for $\delta_{p}^{\max}$ compared with $\delta_{p}^{\min}$. This is because $\delta_{p}^{\max}$ is computed with relatively deterministic values *D_rtt_* and $J_{i}^{-}$. Hence, we can state that it is recommended to use $\delta_{p}^{\max}$ for best performance with our heuristic.

**Figure 11:**
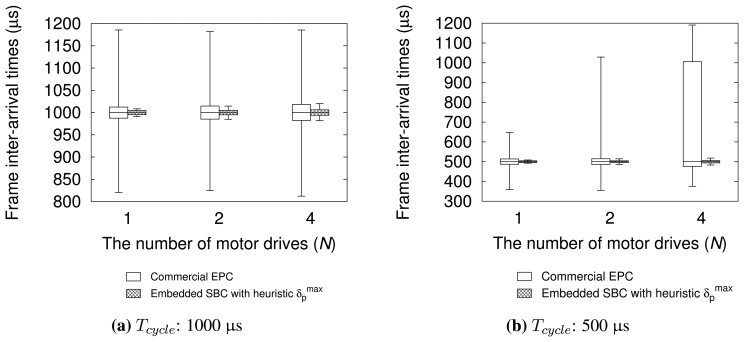
Performance comparison between embedded SBC and commercial controller.

We also compared the performance of embedded SBC and that of a commercial EtherCAT master controller, which has similar hardware specification. All experimental configurations are the same as previous experiments, except for the implementation of motion control task. We used TwinCAT-2 NC PTP runtime to implement a motion application for the commercial controller. Figure 11 shows the performance evaluation results. The results demonstrate that embedded SBC with our heuristic outperforms the commercial controller for all the test configurations used. For *T_cycle_* = 1000 μs, the commercial controller has good performance in average because the 99% of measured samples are located in 24.9 μs, 29.4 μs, and 35.7 μs, which amount to 2%–3% with respect to *T_cycle_* for varying *N*. However, for a smaller control cycle *T_cycle_* = 500 μs, the performance degradation of the commercial controller is noticeable with increasing *N* in terms of absolute difference Δ. By contrast, the evaluation results of our approach show quite stable distribution for all the test configurations. Although the commercial controller used in the experiment may not be fully optimized, the results imply that our approach can achieve performance comparable to a commercial solution.

**Figure 12:**
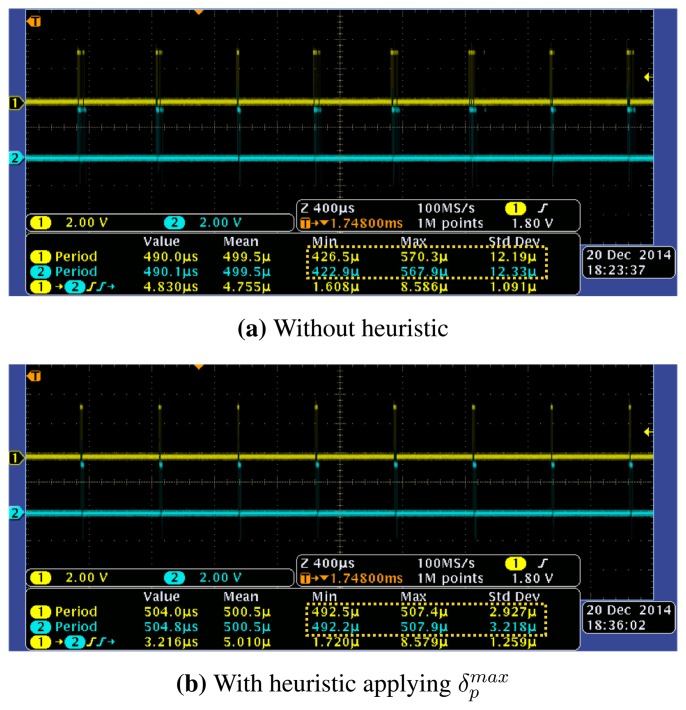
Frame inter-arrival times for different motor drives (Drive 1 and Drive 8) where *T_cycle_* = 500 μs, *N* = 8.

We now consider the usefulness of our approach for guaranteeing the synchronicity among multiple motor drives. For the purpose, we measured the frame inter-arrival times observed at different motor drives by connecting GPIO pins of corresponding motor drives to the probes of oscilloscope. In the experiment, we used embedded SBC for the motion controller. Figure 12 demonstrates the measurement results of $T_{i}^{\text{drv}\left(1\right)}$ and $T_{i}^{\text{drv}\left(8\right)}$ with *T_cycle_* = 500 μs and *N* = 8. The observations from the measurement results are as follows. First, although the measurements were conducted for the first drive and the last drive in the control network, the characteristics of frame inter-arrival times observed at those drives are similar to each other. This means that the communication delays and frame switching overheads are highly deterministic throughout the control network regardless of the relative position of a drive. Second, the inter-arrival times with our heuristic performs similar to the case without heuristic in average. However, the absolute jitter of frame inter-arrival times has been dramatically reduced after applying our heuristic. This characteristic makes it easy to determine a safe clock shift time for accurate clock-based synchronization described in Section 2.2, and thus contributes to higher synchronicity of coordinated motion.

## 5. Conclusions

In modern networked motion control systems, guaranteeing isochronous control is very crucial for higher precision and synchronicity of target motion application. This paper proposed a simple and efficient heuristic approach to guarantee isochronous control by adjusting message transfer phase offset based on pre-runtime analysis. We also made it easy to apply phase offset adjustment code to the auto-generated motion application runtime code by integrating the pre-runtime analysis stubs and user-controlled phase offset with an open-source motion application IDE. Our proposed heuristic can also be effectively used to determine a safe shift time of global clock for accurately coordinated motion of multiple motor drives.

For in-depth and practical evaluation, we conducted extensive measurements for various configurations on a realistic testbed that was constructed from open-source software and COTS hardware platforms. The performance evaluation results show that our heuristic can bound successive actuation jitters within 1%–2% of the control cycle time even on a low-end embedded processor without any kernel-level modification and customized hardware. We also observed that our approach can provide performances comparable to or even better than that of a commercial controller.

In our future research, we will study how to extend our heuristic to support lightweight on-line adjustment. We also plan to extend our model for multiple control tasks with heterogeneous periods to provide higher precision and synchronicity of complex control under heavy interfering workloads such as monitoring and logging.

## Figures and Tables

**Table 1:** Specifications of testbed.

**Hardware**	**Description**	**Software**	**Description**
Industrial PC	Intel i7-620M 2.66 GHz (Single Core enabled.) 2 GB DDR3 Memory, 128 GB SSD Realtek RTL 8139D 100 Mbps Ethernet	Operating Systems Automation Software	Linux 3.8.13, Xenomai 2.6.3 Beremiz 1.1, IgH EtherCAT Master 1.5.2

Embedded SBC	TI AM3358 ARM Cortex-A8 1 GHz 512 MB DDR3 Memory, 2 GB eMMC TI cpsw 100 Mbps Ethernet		

Commercial EPC	Cortex-A8 1 GHz 1 GB DDR3 Memory, 512 MB microSD 100 Mbps Ethernet	Operating Systems Automation Software	MS Windows Embedded Compact 7 TwinCAT 2 NC PTP runtime

EtherCAT Drives	TI AM 3359 ICE board 8 E.A 22 bytes for each drive (RXPDO:11 bytes, TXPDO:11 bytes)	Firmware	TI sysbios sdk 1.0.9

DAQ module	National Instrument USB-6356, 1.25 MS/s/ch, 32 MS memory	N / A	N/A

Oscilloscope	Tektronix DPO 3014, 4 ch, 2.5 GS/s	N/A	N/A

**Table 2:** Measurement results of *J_i_*, $R_{i}^{\text{comp}}$, and $M_{i}^{\text{con}}$ for different controllers.

*Controller*	Embedded SBC												IPC											
*T_cycle_*	1000 μs				500 μs				250 μs				1000 μs				500 μs				250 μs			
*N*	1	2	4	8	1	2	4	8	1	2	4	8	1	2	4	8	1	2	4	8	1	2	4	8
*J_i_* (μs)																								
	
avg.	0.0	0.0	0.0	0.0	0.0	0.0	0.0	0.0	0.0	0.0	0.0	0.0	0.0	0.0	0.0	0.0	0.0	0.0	0.0	0.0	0.0	0.0	0.0	0.0
	
min.	_−_16.9	_−_22.0	_−_19.4	_−_20.3	_−_15.3	_−_15.8	_−_15.2	_−_14.0	_−_10.6	_−_9.8	_−_7.7	_−_7.7	_−_6.8	_−_9.2	_−_10.6	_−_9.7	_−_10.0	_−_9.6	_−_10.6	_−_8.7	_−_9.8	_−_9.3	_−_9.9	_−_10.5
	
max.	24.8	32.4	31.2	29.3	22.9	18.5	20.8	17.5	12.5	12.2	11.4	9.4	6.2	9.7	11.3	9.6	10.2	10.1	10.5	9.4	9.4	10.5	9.9	10.2
	
st.d (*σ*)	1.5	1.6	1.8	2.1	1.2	1.3	1.4	1.7	0.8	0.9	0.8	1.1	1.5	1.8	1.5	1.8	1.4	1.9	1.6	1.8	1.7	1.8	1.7	1.8
	
diff. (Δ)	41.7	54.4	50.6	49.6	38.2	34.3	36.0	31.5	23.1	22.0	19.1	17.1	13.0	18.9	21.9	19.3	20.2	19.7	21.1	18.1	19.2	19.8	19.8	20.7

$R_{i}^{\text{comp}}$ (μs)																								
	
avg.	54.8	78.1	123.8	226.3	52.7	76.5	120.8	214.8	51.8	74.8	120.1	211.4	32.3	43.5	67.7	119.7	32.2	43.7	66.6	119.5	31.9	42.9	67.2	118.6
	
min.	49.0	71.5	116.4	207.3	48.3	71.7	116.2	204.4	48.3	71.1	115.9	203.8	30.2	41.3	62.0	115.7	30.4	40.5	62.6	115.4	30.0	40.5	61.6	114.1
	
max.	150.3	174.3	250.7	407.3	115.1	137.8	207.4	310.0	83.7	107.6	159.6	235.6	38.2	51.8	74.3	126.4	38.4	50.2	73.2	126.7	38.8	50.8	74.2	125.4
	
st.d (*σ*)	3.7	4.2	5.9	11.7	3.1	3.6	5.3	8.2	2.1	2.2	2.7	4.0	0.6	0.7	0.7	1.9	0.5	0.5	0.5	0.6	0.4	0.5	0.5	0.4
	
diff. (Δ)	101.7	102.8	134.3	200.0	68.8	66.1	91.2	105.6	40.4	36.5	43.7	31.8	8.0	10.5	12.3	10.7	8.0	9.7	10.6	11.3	8.8	10.3	12.6	11.3

$M_{i}^{\text{con}}$ (μs)																								
	
avg.	54.8	78.1	123.8	226.3	52.7	76.5	120.8	214.8	51.8	74.8	120.1	211.4	32.3	43.5	67.7	119.7	32.2	43.7	66.6	119.5	31.9	42.9	67.2	118.6
	
min.	36.9	64.2	112.7	206.9	38.3	65.9	110.9	201.8	43.2	68.0	112.7	203.1	25.4	35.0	58.3	111.3	23.1	34.5	57.0	111.9	22.9	34.3	57.4	105.2
	
max.	150.3	199.1	279.6	429.0	132.2	154.2	215.1	324.3	93.0	115.2	167.6	235.8	42.2	53.7	83.9	132.7	43.4	56.3	78.5	131.5	42.0	56.1	77.6	131.3
	
st.d (*σ*)	4.8	5.3	7.0	13.0	4.0	4.4	6.1	9.4	2.6	2.8	3.3	4.5	1.6	1.9	1.7	1.9	1.5	1.9	1.7	1.9	1.8	1.9	1.8	1.8
	
diff. (Δ)	113.4	134.9	166.9	222.1	93.9	88.3	104.2	122.5	49.8	47.2	54.9	32.7	16.8	18.7	25.6	21.4	20.3	21.8	21.5	19.6	19.1	21.8	20.2	26.1

**Table 3:** Normalized phase offset factor *δ_p_* for Embedded SBC and IPC.

*Controller*	***N***	1			2			4			8		

	***δ p* (%)**	$\delta_{p}^{\text{min}}$	$\delta_{p}^{\mathrm{med}}$	$\delta_{p}^{\max}$	$\delta_{p}^{\min}$	$\delta_{p}^{\mathrm{med}}$	$\delta_{p}^{\max}$	$\delta_{p}^{\min}$	$\delta_{p}^{\mathrm{med}}$	$\delta_{p}^{\max}$	$\delta_{p}^{\min}$	$\delta_{p}^{\mathrm{med}}$	$\delta_{p}^{\max}$
Embedded SBC	*T_cycle_* = 1000 μs	16	57	97	20	58	96	28	62	96	43	69	94

	*T_cycle_* = 500 μs	27	61	95	31	63	94	44	68	92	65	77	89

	*T_cycle_* = 250 μs	38	65	92	47	69	91	68	78	88	**N/A**	**N/A**	**N/A**

IPC	*T_cycle_* = 1000 μs	5	52	98	6	52	98	9	53	96	14	55	95

	*T_cycle_* = 500 μs	9	53	96	12	54	95	16	55	93	27	59	90

	*T_cycle_* = 250 μs	17	55	93	23	58	92	32	60	88	53	67	80

**Table 4:** Comparison of frame inter-arrival times for different controller configurations.

***Cont.***	**Embedded SBC**								**IPC**							

***Tcycle***	**1000μs**				**500 μs**				**1000 μs**				**500 μs**			

***N***	**1**	**2**	**4**	**8**	**1**	**2**	**4**	**8**	**1**	**2**	**4**	**8**	**1**	**2**	**4**	**8**
Frame inter-arrival time, without heuristic																
	
avg.	1000.0	1000.0	1000.0	1000.0	500.0	500.0	500.0	500.0	1000.0	1000.0	1000.0	1000.0	500.0	500.0	500.0	500.0
	
min.	927.4	909.2	898.8	860.9	439.5	436.1	425.5	413.0	989.2	987.2	986.5	985.2	487.3	488.9	487.8	486.4
	
max.	1092.5	1120.3	1154.3	1180.9	578.7	577.0	589.4	620.9	1011.2	1013.5	1012.4	1014.3	514.9	513.0	514.1	515.3
	
Low 0.5%	983.6	981.0	975.4	960.2	486.3	483.2	481.0	467.5	993.7	993.0	993.3	991.4	494.4	493.1	493.9	492.2
	
Low 99.5%	1018.3	1021.6	1028.1	1048.8	515.9	519.3	523.2	542.4	1006.5	1006.9	1006.8	1008.4	506.4	506.5	506.4	507.5
	
st.d (*σ*)	6.5	6.9	9.3	14.4	5.4	6.1	7.2	14.0	2.3	2.4	2.5	3.3	2.0	2.4	2.2	2.8
	
diff. (Δ)	165.1	211.1	255.5	320.0	139.2	140.9	163.9	207.9	22.0	26.3	25.9	29.1	27.6	24.1	26.3	28.9
	
*ϵ* 1%	74,334	92,133	233,650	1,112,919	1,406,008	1,588,274	1,794,865	2,376,029	52	234	156	3,211	99,161	185,027	131,175	298,757
	
*ϵ* 10%	0	3	17	1275	81	683	4137	13403	0	0	0	0	0	0	0	0

Frame inter-arrival time, with heuristic applying *δ max*																
	
avg.	1000.0	1000.0	1000.0	1000.0	500.0	500.0	500.0	500.0	1000.0	1000.0	1000.0	1000.0	500.0	500.0	500.0	500.0
	
min.	991.0	984.9	982.0	979.9	492.2	485.5	483.2	479.0	987.9	989.6	988.3	981.0	487.8	489.4	488.5	483.0
	
max.	1008.3	1014.5	1020.3	1021.7	508.8	514.5	518.5	521.5	1010.5	1010.0	1010.5	1018.5	512.8	510.9	511.9	516.7
	
Low 0.5%	995.2	994.9	993.8	991.9	495.9	495.8	493.9	493.4	993.3	993.3	993.4	991.1	493.2	493.3	493.4	492.6
	
Low 99.5%	1004.7	1004.9	1006.2	1007.9	504.4	504.6	505.5	506.8	1006.4	1006.1	1006.2	1008.5	506.5	506.1	506.1	507.2
	
st.d (*σ*)	2.0	3.1	3.1	4.2	1.7	1.8	2.5	2.8	2.2	2.2	2.3	3.1	2.2	2.2	2.3	2.6
	
diff. (Δ)	17.3	29.6	38.3	41.8	16.6	29.0	35.3	42.5	22.6	20.4	22.2	37.5	25.0	21.5	23.4	33.7
	
*ϵ* 1%	0	13	102	841	5,765	10,725	127,923	260,307	13	5	23	3,813	176,025	147,529	164,685	234,918
	
*ϵ* 10%	0	0	0	0	0	0	0	0	0	0	0	0	0	0	0	0
